# Editorial: Bayesian Inference and AI

**DOI:** 10.3389/fdata.2022.934362

**Published:** 2022-06-30

**Authors:** Niansheng Tang, Catherine Liu, Jian Qing Shi, Yangxin Huang

**Affiliations:** ^1^Department of Statistics, Yunnan University, Kunming, China; ^2^Department of Applied Mathematics, Hong Kong Polytechnic University, Kowloon, Hong Kong SAR, China; ^3^Department of Statistics and Data Science, Southern University of Science and Technology, Shenzhen, China; ^4^Department of Epidemiology & Biostatistics, University of South Florida, Tampa, FL, United States

**Keywords:** Bayesian inference, artificial intelligence, Markov chain Monte Carlo, posterior expectation, Wasserstein Impact Measure, linkage tailoring, text debiasing

The Bayesian theory (Bayes, 1763), which describes the probability of an event given some prior knowledge, has been the bedrock for modern Bayes inference and statistics, and the Markov chain Monte Carlo (MCMC) algorithm (Hastings, 1970) has been the dominating force of the Bayesian inference methods since its inception. However, the big data era has witnessed paradigm-shifting developments in a variety of Bayesian fronts, including theories, methodologies, computational techniques, and applications, many of which have been particularly designed to respond to emerging artificial intelligence (AI) and data science.

Though much progress has been achieved in developing the interface between Bayesian inference and AI, a large number of important issues, such as how to design efficient Bayesian computational algorithms and modeling and inferential methods so that they can be readily adaptive to the evolving AI technology, remain to be addressed and solved. As a continuing effort for understanding the interface of Bayesian inference and AI, and their joint applications in a variety of biomedical, biological, engineering, and data science fields, we have presented this special issue which features the work of several prominent authors, in theory, methods, algorithms, and applications.

Zhang et al. discussed how to use Bayesian inference to estimate the variance and scale parameters with conjugate and non-informative priors under the posterior expected Stein's loss (PESL). Intensive simulations exemplified the theoretical findings and the feasibility of the proposal, and their method was applied to analyze the S&P 500 monthly simple returns.

Changing gear, Huang et al. further developed a new class of Bayesian multivariate joint models with a skewed normal distribution for modeling longitudinal and time-to-event data. Their methods could cope with correlated multiple longitudinal exposures, adjust departures from normality, and tailor linkage when specifying a survival process. The methods were applied to a diabetes study.

Rad et al. proposed a new Bayesian directional data model for the skew-rotationally-symmetric Fisher-von Mises-Langevin (FvML) distribution. A pivotal building block was specified for the prior distributions so that the impact of priors could be quantified via the Wasserstein Impact Measure (WIM), which may facilitate practitioners' choices in implementation. A modified Gibbs and slice sampling algorithm was also designed for generating posterior samples.

Finally, Hu et al. developed a new algorithm to solve a bias problem in job advertisements. The new algorithm could help “debias” the texts by offering alternative and more appropriate words that may be most closely related to the original intent. Their methods could see a variety of human resources applications, ranging from the development of job advertisements to algorithm-assisted screening of job applications.

We believe that the papers published in this special issue demonstrate the breadth and depth of the development of Bayesian theory, methods, computational techniques, and applications in response to AI, and will spark continued research in these emerging fields. We are very grateful to the authors for their important contributions.

## Author Contributions

All authors revised this manuscript. All authors contributed to the article and approved the submitted version.

## Conflict of Interest

The authors declare that the research was conducted in the absence of any commercial or financial relationships that could be construed as a potential conflict of interest.

## Publisher's Note

All claims expressed in this article are solely those of the authors and do not necessarily represent those of their affiliated organizations, or those of the publisher, the editors and the reviewers. Any product that may be evaluated in this article, or claim that may be made by its manufacturer, is not guaranteed or endorsed by the publisher.

## References

[B1] Bayes T. (1763). An essay towards solving a problem in the doctrine of hances. Philos. Trans. 53, 370–418. 1857193

[B2] Hastings W. K. (1970). Monte Carlo sampling methods using Markov Chains and their applications. Biometrika 57, 97–109.

